# Sub-Optimality of the Early Visual System Explained Through Biologically Plausible Plasticity

**DOI:** 10.3389/fnins.2021.727448

**Published:** 2021-09-15

**Authors:** Tushar Chauhan, Timothée Masquelier, Benoit R. Cottereau

**Affiliations:** ^1^Centre de Recherche Cerveau et Cognition, Université de Toulouse, Toulouse, France; ^2^Centre National de la Recherche Scientifique, Toulouse, France

**Keywords:** vision, cortex, plasticity, suboptimality, Independent Component Analysis, Sparse Coding, STDP, natural statistics

## Abstract

The early visual cortex is the site of crucial pre-processing for more complex, biologically relevant computations that drive perception and, ultimately, behaviour. This pre-processing is often studied under the assumption that neural populations are optimised for the most efficient (in terms of energy, information, spikes, etc.) representation of natural statistics. Normative models such as Independent Component Analysis (ICA) and Sparse Coding (SC) consider the phenomenon as a generative, minimisation problem which they assume the early cortical populations have evolved to solve. However, measurements in monkey and cat suggest that receptive fields (RFs) in the primary visual cortex are often noisy, blobby, and symmetrical, making them sub-optimal for operations such as edge-detection. We propose that this suboptimality occurs because the RFs do not emerge through a global minimisation of generative error, but through locally operating biological mechanisms such as spike-timing dependent plasticity (STDP). Using a network endowed with an abstract, rank-based STDP rule, we show that the shape and orientation tuning of the converged units are remarkably close to single-cell measurements in the macaque primary visual cortex. We quantify this similarity using physiological parameters (frequency-normalised spread vectors), information theoretic measures [Kullback–Leibler (KL) divergence and Gini index], as well as simulations of a typical electrophysiology experiment designed to estimate orientation tuning curves. Taken together, our results suggest that compared to purely generative schemes, process-based biophysical models may offer a better description of the suboptimality observed in the early visual cortex.

## Introduction

The human visual system processes an enormous throughput of sensory data in successive operations to generate percepts and behaviours necessary for biological functioning (Anderson et al., 2005; Raichle, 2010). Computations in the early visual cortex are often explained through unsupervised normative models which, given an input dataset with statistics similar to our surroundings, carry out an optimisation of criteria such as energy consumption and information-theoretic efficiency (Olshausen and Field, 1996; Bell and Sejnowski, 1997; van Hateren and van der Schaaf, 1998; Hoyer and Hyvärinen, 2000; Zhaoping, 2006; Bruce et al., 2016). While such approaches could explain why many properties of the early visual system are closely related to characteristics of natural scenes (Olshausen and Field, 1996; Bell and Sejnowski, 1997; Lee and Seung, 1999; Geisler, 2008; Hunter and Hibbard, 2015; Beyeler et al., 2019), they are not equipped to answer questions such as how cortical structures which support complex computational operations implied by such optimisation may emerge, how these structures adapt, even in adulthood (Wandell and Smirnakis, 2010; Hübener and Bonhoeffer, 2014), and why some neurones possess receptive fields (RFs) which are sub-optimal in terms of information processing (Jones and Palmer, 1987; Ringach, 2002).

It is now well established that locally driven synaptic mechanisms such as spike-timing dependent plasticity (STDP) are natural processes which play a pivotal role in shaping the computational architecture of the brain (Markram et al., 1997; Delorme et al., 2001; Caporale and Dan, 2008; Larsen et al., 2010; Masquelier, 2012; Brito and Gerstner, 2016). Indeed, locally operating implementations of generative schemes have been shown to be closer to biological measurements (see, e.g., Rozell et al., 2008). Therefore, it is only natural to hypothesise that locally operating, biologically plausible models of plasticity must offer a better description of RFs in early visual cortex. However, such line of reasoning leads to the obvious question: what exactly constitutes a “better description” of a biological system, and more specifically, the early visual cortex. Here, we use a series of criteria spanning across electrophysiology, information theory, and machine learning, to investigate how descriptions of early visual RFs provided by a local, abstract STDP model compare to biological data from the macaque. We also compare these results to two classical, and important normative schemes – Independent Component Analysis (ICA), and Sparse Coding (SC). Our results demonstrate that a local process-based model of experience-driven plasticity may be better suited to capturing the RFs of simple-cells, thus suggesting that biological preference does not always concur with forms of global, generative optimality.

More specifically, we show that STDP units are able to better capture the characteristic sub-optimality in RF shape reported in literature (Jones and Palmer, 1987; Ringach, 2002), and their orientation tuning closely matches measurements in the macaque primary visual cortex (V1) (Ringach et al., 2002). Taken together, our findings suggest that while the information carrying capacity of an STDP ensemble is not optimal when compared to generatively optimal schemes, it is precisely this sub-optimality which may make process-based, local models more suited for describing the initial stages of sensory processing.

## Materials and Methods

### Dataset

**FIGURE 1:**
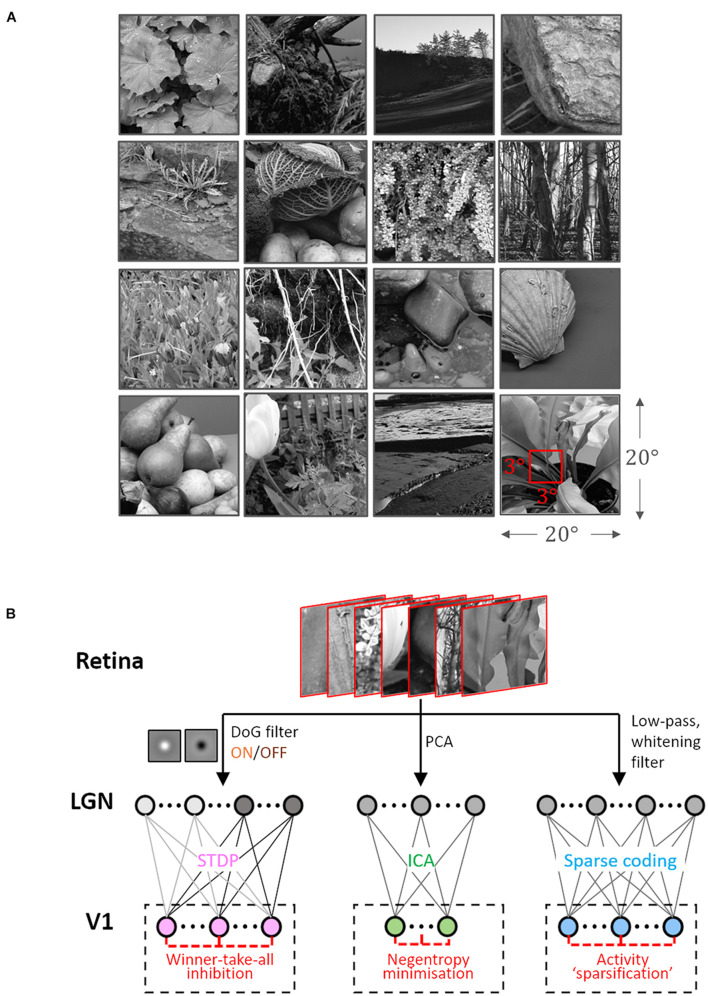
Dataset and the computational pipeline. **(A)** Training data. The Hunter–Hibbard dataset of natural images was used. The images in the database have a 20°×20° field of view. Patches of size 3°×3° were sampled from random locations in the images (overlap allowed). The same set of 100,000 randomly sampled patches was used to train three models: Spike-timing dependent plasticity (STDP), Independent Component Analysis (ICA), and Sparse Coding (SC). **(B)** Modelling the early visual pathway. Three representative stages of early visual computation were captured by the models: retinal input, processing in the lateral geniculate nucleus (LGN), and the activity of early cortical populations in the primary visual cortex (V1). Each input patch represented a retinal input. This was followed by filtering operations generally associated with the LGN, such as decorrelation and whitening. Finally, the output from the LGN units/filters was connected to the V1 population through all-to-all (dense) plastic synapses which changed their weights during learning. Each model had a specific optimisation strategy for learning: the STDP model relied on a local rank-based Hebbian rule, ICA minimised mutual information (approximated by the negentropy), and SC enforced sparsity constraints on V1 activity. DoG, difference of Gaussian; PCA, Principal Component Analysis.

The Hunter–Hibbard dataset of natural images was used (Hunter and Hibbard, 2015) for training. It is available under the MIT license at https://github.com/DavidWilliamHunter/Bivis, and consists of 139 stereoscopic images of natural scenes captured using a realistic acquisition geometry and a 20° field of view. Only images from the left channel were used, and each image was resized to a resolution of 5 px/° along both horizontal and vertical directions. Inputs to all encoding schemes were 3×3° patches (i.e., 15×15 px) sampled randomly from the dataset (Figure 1A).

### Encoding Models

Samples from the dataset were used to train and test three models corresponding to the ICA, SC, and STDP encoding schemes. Each model consisted of three successive stages (Figure 1B). The first stage represented retinal activations. This was followed by a pre-processing stage implementing operations which are typically associated with processing in the lateral geniculate nucleus (LGN), such as whitening and decorrelation. In the third stage, LGN output was used to drive a representative V1 layer.

During learning, 10^5^ patches (3×3°) were randomly sampled from the dataset to simulate input from naturalistic scenes. In this phase, the connections between the LGN and V1 layers were plastic, and modified in accordance with one of the three encoding schemes. Care was taken to ensure that the sequence of inputs during learning was the same for all three models. After training, the weights between the LGN and V1 layers were no longer allowed to change. The implementation details of the three models are described below.

#### Sparse Coding

Sparse Coding algorithms are based on energy-minimisation, which is typically achieved by a “sparsification” of activity in the encoding population. We used a now-classical SC scheme proposed by Olshausen and Field (1996, 1997). The pre-processing in this scheme consists of an initial whitening of the input using low pass filtering, followed by a trimming of higher frequencies. The latter was employed to counter artefacts introduced by high frequency noise, and the effects of sampling across a uniform square grid. In the frequency domain the pre-processing filter was given by a zero-phase kernel:


(1) $$\begin{array}{c} H\left(f\right)=f\cdot e^{-{\left(\frac{f}{f_{0}}\right)}^{4}} \end{array}$$

Here, *f*_0_ = 10 cycles/° is the cut-off frequency. The outputs of these LGN filters were then used as inputs to the V1 layer composed of 225 units (3° × 3° RF at 5 px/°). The total number of weights in the model was 50,625. Retinal projections of the converged RFs were recovered by an approximate reverse-correlation algorithm (Ringach, 2002; Ringach and Shapley, 2004) derived from a linear-stability analysis of the SC objective function about its operating point. The RFs (denoted as columns of a matrix, say ξ) were given by:


(2) $$\begin{array}{c} \xi =\text{A}{\left[{\text{A}}^{T}\text{A}+\lambda S"\left(0\right)\text{I}\right]}^{-1} \end{array}$$

Here, **A** is the matrix containing converged sparse components as column vectors, λ is the regularisation parameter (for the reconstruction, it is set to 0.14σ, where σ^2^ is the variance in the input dataset), and *S*(*x*) is the shape-function for the prior distribution of the sparse coefficients [this implementation uses *log* (1 + *x*^2^)].

#### Independent Component Analysis

Independent Component Analysis algorithms are based on the idea that the activity of an encoding ensemble must be as information-rich as possible. This typically involves a maximisation of mutual information between the retinal input and the activity of the encoding ensemble. We used a classical ICA algorithm called *fastICA* (Hyvärinen and Oja, 2000) which achieves this through an iterative estimation of input negentropy. The pre-processing in this implementation was performed using a truncated Principal Component Analysis (PCA) transform ($\tilde{d}=150$ components were used), leading to low-pass filtering and local decorrelation akin to centre-surround processing reported in the LGN. The model fit a total of 33,750 weights. If the input patches are denoted by the columns of a matrix (say **X**), the LGN activity **L** can be written as:


(3) $$\begin{array}{c} \text{L}={\tilde{\text{U}}}^{T}{\text{X}}_{C} \end{array}$$

Here, **X**_*C*_ = **X**−⟨**X**⟩ and $\tilde{\text{U}}$ is a matrix composed of the first $\tilde{d}\;\left(=150\right)$ principal components of **X**_*C*_. The activity of these LGN filters was then used to drive the ICA V1 layer consisting of 150 units, with its activity **Σ** being given by:


(4) $$\begin{array}{c} \Sigma =\text{WL} \end{array}$$

Here, **W** is the un-mixing matrix which is optimised during learning. The recovery of the RFs for ICA was relatively straight forward, as, in our implementation, they were assumed to be equivalent to the filters which must be applied to a given input to generate the corresponding V1 activity. The RFs (denoted as columns of a matrix, say ξ) were given by:


(5) $$\begin{array}{c} \xi =\tilde{\text{U}}{\text{W}}^{T}+\left\langle X\right\rangle \end{array}$$

#### Spike-Timing Dependent Plasticity

Spike-timing dependent plasticity is a biologically observed, Hebbian-like learning rule which relies on local spatiotemporal patterns in the input. We used a feedforward model based on an abstract rank-based STDP rule (Chauhan et al., 2018). The pre-processing in the model consisted of half-rectified ON/OFF filtering using difference-of-Gaussian kernels based on the properties of magno-cellular LGN cells. The outputs of these filters were converted to relative first-spike latencies using a monotonically decreasing function (1/*x* was used), and only the earliest 10% spikes were allowed to propagate to V1 (Delorme et al., 2001; Masquelier and Thorpe, 2007). For each iteration, spikes within this 10% window were used to drive an unsupervised network of 225 integrate-and-fire neurones. The membrane potential of a V1 neurone was given by:


(6) $$u\left(t\right)=\text{H}\left(\theta -u\right)\sum_{i\in LGN}w_{i}\left(t\right)\delta \left(t-t_{i}\right)$$

Here, *t*_*i*_ denotes the latency of the *i*-th pre-synaptic neuron, H is the Heavisde function, and θ is the spiking threshold. During learning, changes in the synaptic weights between LGN and V1 were governed by a rank-based, simplified version of the STDP rule proposed by Gütig et al. (2003). After each iteration, the change (Δ*w*) in the weight (*w*) of a given synapse was given by:


(7) $$\begin{array}{c} \Delta w=\left\{ \begin{array}{c} -\alpha^{-}\cdot {\left(w-w_{min}\right)}^{\mu^{-}}\text{K}\left(t,\tau^{-}\right),t\leq 0\\ \alpha^{+}\cdot {\left(w_{max}-w\right)}^{\mu^{+}}\text{K}\left(t,\tau^{+}\right),t>0 \end{array}\right. \end{array}$$

Here, Δ*t* is the difference between the post- and pre-synaptic spike times, the constants α^±^ describe the learning rates for long-term potentiation (LTP) and depression (LTD), respectively, μ^±^ ∈ [0, 1] characterise the non-linearity of the multiplicative updates, K is a windowing function, and τ^±^ are the time-scales for LTP and LTD windows. Note that *w* is soft-bound such that *w* ∈ (*w*_*min*_, *w*_*max*_). The model used *w*_*min*_ = 0 (thalamocortical connections are known to be excitatory in nature), and *w*_*max*_ = 1. Since the intensity-to-latency conversion operates on an arbitrary time-scale, weight updates were based on the spike-order rather than precise spike-timing (rank-based). This meant that the window for LTP (τ^+^) was variable and driven by the first 10% thalamic spikes, while the window for LTD (τ^−^) was theoretically infinite. During updates, the weight was increased if a presynaptic spike occurred before the postsynaptic spike (causal firing), and decreased if it occurred after the post-synaptic spike (acausal firing). The learning rates were α^+^ = 5 × 10^−3^ and α^−^ = 0.75 × α^+^, and the nonlinearities were μ^+^ = 0.65 and μ^−^ = 0.05. The model has previously been shown to be robust to both internal and external noise, and the parameter values were chosen from a range which best approximates the behaviour of the model under a realistic, V1-like regime (Chauhan et al., 2018). The neural population was homogeneous, with each neuron described by the exact same set of parameters.

During each iteration of learning, the population followed a winner-take-all inhibition rule wherein the firing of one neurone reset the membrane potentials of all other neurones. A total of 50,625 weights were fit by the model. After learning, this inhibition was no longer active and multiple units were allowed to fire for each input – allowing us to measure the behaviour of the network during testing. This also renders the model feed-forward only, making it comparable to SC and ICA. The RFs of the converged neurones were recovered using a linear approximation. If *w_i_* denotes the weight of the synapse connecting a given neurone to the *i*th LGN filter with RF **ψ_i_**, the RF **ξ** of the neurone was given by:


(8) $$\xi =\sum_{i\in LGN}w_{i}\psi_{\text{i}}$$

### Evaluation Metrics

#### Gabor Fitting

Linear approximations of RFs obtained by each encoding strategy were fitted using 2-D Gabor functions. This is motivated by the fact that all the encoding schemes considered here lead to linear, simple-cell-like RFs. In this case, the goodness-of-fit parameter (*R*^2^) provides an intuitive measure of how Gabor-like a given RF is. The fitting was carried out using an adapted version of the code available at https://uk.mathworks.com/matlabcentral/fileexchange/60700-fit2dgabor-data-options (Ecke et al., 2021).

#### Frequency-Normalised Spread Vector

The shape of the RFs approximated by each encoding strategy was characterised using frequency-normalised spread vectors (FSVs) (Ringach, 2002; Chauhan et al., 2018). For a RF fitted by a Gabor-function with sinusoid carrier frequency *f* and envelope size σ = [σ_*x*_ σ_*y*_]^*T*^, the FSV is given by:


(9) $$\begin{array}{c} {\left[n_{x}n_{y}\right]}^{T}={\left[\sigma_{x}\sigma_{y}\right]}^{T}f \end{array}$$

While *n_x_* provides an intuition of the number of cycles in the RF, *n_y_* is a cycle-adjusted measure of the elongation of the RF perpendicular to the direction of sinusoid propagation. The FSV serves as a compact, intuitive descriptor of the RF shape-invariance to affine operations such as translation, rotation, and isotropic scaling.

#### Orientation Tuning

Orientation tuning curves (OTCs) were estimated by presenting each unit in each model with noisy oriented sine-wave grating (SWG) stimuli at its preferred frequency. The orientation was sampled in steps of 2° in the interval [0°, 180]°. For each orientation, the activity was averaged over phase values uniformly sampled in the interval [0°, 360°] using a step-size of 5°. The bandwidth of an OTC was taken as its half-width at $1/\sqrt{2}$ of the peak response (Ringach et al., 2002). The whole process was repeated 100 times, and a bootstrap procedure was used to determine 95% confidence intervals.

#### Fisher Information

The information content in the activity of the converged units was quantified by using approximations of the Fisher information (FI, denoted here by the symbol *J*). If **x** = {*x*_1_, *x*_2_, *x*_3_, *x*_*N*_} is a random variable describing the activity of an ensemble of *N* independent units, the FI of the population with respect to a parameter θ is given by:


(10) $$\begin{array}{c} J\left(\theta \right)=\sum_{i=1}^{N}E{\left[{\left\{\frac{\partial }{\partial x}\ln P\left(x_{i}|\theta \right)\right\}}^{2}\right]}_{P\left(x_{i}|\theta \right)} \end{array}$$

Here, *E*[.]_*P*(*x*_*i*_|θ)_ denotes expectation value with respect to the firing-state probabilities of the *i*th neurone in response to the stimuli corresponding to parameter value θ. In our simulations, θ was the orientation (defined as the direction of travel) of a set of SWGs with additive Gaussian noise, and was sampled at intervals of 4° in the range [0°, 180°). The SWGs were presented at frequency of 1.25 cycles/visual degree, and the responses were calculated by averaging over 8 evenly spaced phase values in [0°, 360°). This effectively simulated a drifting grating design within the constraints of the computational models. Each simulation was repeated 100 times and a jackknife procedure was used to estimate 95% confidence intervals. Noise was added such that the signal-to-noise ratio (SNR) varied between −6 and 6 dB in steps of 1 dB.

#### Decoding Using a Linear Classifier

In addition to FI approximations, we also used a linear decoder on the population responses obtained in the FI simulations. The decoder was an error-correcting output codes model composed of binary linear-discriminant classifiers configured in a one-vs.-all scheme. Similar to the FI experiment, ground-truth values of the orientation at intervals of 4° in the range [0°, 180°) were used as the class labels, and the activity generated by the corresponding SWG stimuli with added Gaussian noise was used as the training/testing data. The SWGs were presented at a frequency of 1.25 cycles/visual degree, and the responses were calculated by averaging over 8 evenly spaced phase values in [0°, 360°). Each simulation was repeated 100 times, each time with five-fold validation. A jackknife procedure was used to estimate 95% confidence intervals.

#### Post-convergence Threshold Variation in STDP

To test how post-learning changes in the threshold affect the specificity of a converged network, we tested an STDP network trained using a threshold θ_training_ by increasing or decreasing its threshold (to say, θ_testing_) and presenting it with SWGs (same stimuli as the ones used to calculate the FI). We report the results of seven simulations where the relative change in threshold was given by 25% increments/decrements, i.e.:


(11) $$\begin{array}{c} \frac{\theta_{\text{testing}}-\theta_{\text{training}}}{\theta_{\text{training}}}=\left\{0,\pm 0.25,\pm 0.50,\pm 0.75\right\} \end{array}$$

#### Kullback–Leibler Divergence

For each model, we estimated probability density functions (pdfs) over parameters such as the FSVs and the population bandwidth. To quantify how close the model pdfs were to those estimated from the macaque data, we employed the Kullback–Leibler (KL) divergence. KL divergence is a directional measure of distance between two probability distributions. Given two distributions *P* and *Q* with corresponding probability densities *p* and *q*, the KL divergence (denoted *D*_*KL*_) of *P* from *Q* is given by:


(12) $$\begin{array}{c} D_{KL}\left(P||Q\right)=\int_{\Omega }p\left(\text{x}\right)\log_{2}\left(\frac{p\left(x\right)}{q\left(x\right)}\right)d\text{x} \end{array}$$

Here, **Ω** is the support of the distribution *Q*. In our analysis, we considered the reference distribution *p* as a pdf estimated from the macaque data, and *q* as the pdf (of the same variable) estimated using ICA, SC, or STDP. In this case, KL divergence lends itself to a very intuitive interpretation: it can be considered as the additional bandwidth (in bits) which would be required if the biological variable were to be encoded using one of the three computational models. Note that *P* and *Q* may be multivariate distributions.

#### Sparsity: Gini Index

The sparseness of the encoding was evaluated using the Gini index (GI). GI is a measure which characterises the deviation of the population-response from a uniform distribution of activity across the samples. Formally, the GI (denoted here as Λ) is given by:


(13) $$\begin{array}{c} \Lambda \left(x\right)=1-2\int_{0}^{1}L\left(F\right)dF \end{array}$$

Here *L* is the Lorenz function defined on the cumulative probability distribution *F* of the neural activity (say, **x**). GI is 0 if all units have the same response and tends to 1 as responses become sparser (being equal to 1 if only 1 unit responds, while others are silent). It is invariant to the range of the responses within a given sample, and robust to variations in sample-size (Hurley and Rickard, 2009). We defined two variants of the GI which measure the spatial (Λ_s_) and temporal sparsity (Λ_t_) of an ensemble of encoders. Given a sequence of *M* inputs to an ensemble of *N* neurones, the spatial sparsity of the ensemble response to the *m*th stimulus is given by:


(14) $$\begin{array}{c} \Lambda_{\text{S}}\left(m\right)=\Lambda \left(\left\{x_{m}^{1},x_{m}^{2},\ldots ,x_{m}^{N}\right\}\right) \end{array}$$

Here, $x_{m}^{n}$ denotes the activity of the *n*th neurone in response to the *m*th input. Similarly, the temporal sparsity of the *n*th neurone over the entire sequence of inputs is given by:


(15) $$\begin{array}{c} \Lambda_{\text{T}}\left(n\right)=\Lambda \left(\left\{x_{1}^{n},x_{2}^{n},\ldots ,x_{M}^{n}\right\}\right) \end{array}$$

### Code

The code for ICA was written in python using the sklearn library which implements the classical *fastICA* algorithm. The code for SC was based on the C++ and Matlab code shared by Prof. Bruno Olshaussen. The STDP code was based on a previously published binocular-STDP algorithm available at https://senselab.med.yale.edu/ModelDB/showmodel.cshtml?model=245409#tabs-1.

## Results

We used an abstract model of the early visual system with three representative stages: retinal input, LGN processing, and V1 activity (Figure 1B). To simulate retinal activity corresponding to natural inputs, patches of size 3° × 3° (visual angles) were sampled randomly from the Hunter–Hibbard database (Hunter and Hibbard, 2015) of natural scenes (Figure 1A). 10^5^ patches were used to train models corresponding to three encoding schemes: ICA, SC, and STDP. Each model used a specific procedure for implementing the LGN processing and learning the synaptic weights between the LGN and V1 (see Figure 1B and section “Materials and Methods”).

### Receptive Field Symmetry

**FIGURE 2:**
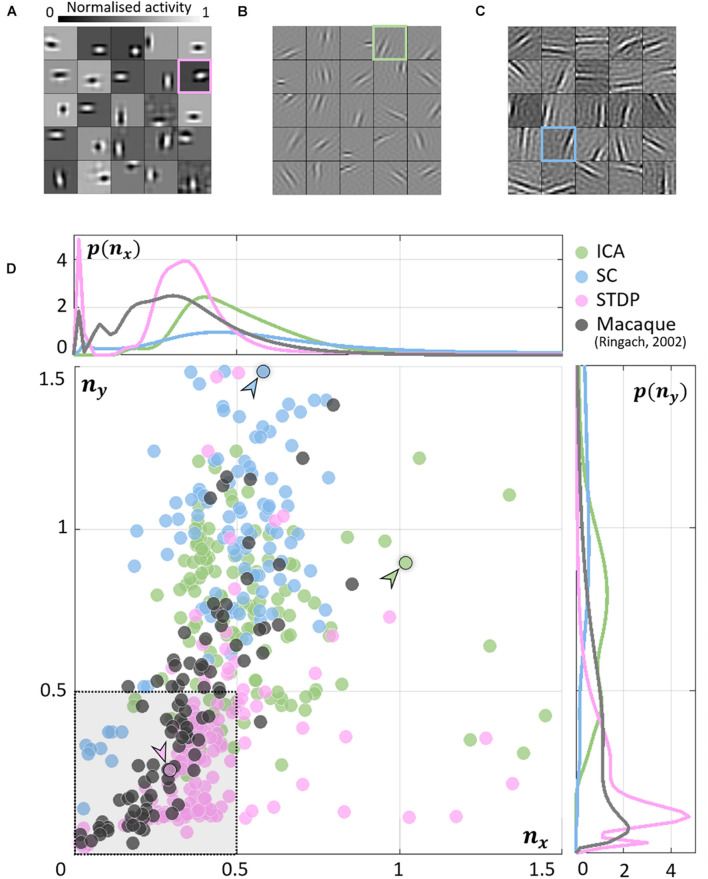
Receptive field (RF) shape. **(A–C)** RFs of neurones randomly chosen from the three converged populations. The STDP population is shown in **(A)**, ICA in **(B)**, and SC in **(C)**. **(D)** Frequency-scaled spread vectors (FSVs). FSV is a compact metric for quantifying RF shape. *n*_*x*_ is proportional to the number of lobes in the RF, *n*_*y*_ is a measure of the elongation of the RF, and values near zero characterise symmetric, often blobby RFs. The FSVs for STDP (pink), ICA (green), and SC (blue), are shown with data from macaque V1 (black) (Ringach, 2002). Measurements in macaque simple-cells tend to fall within the square bound by 0.5 along both axes (shaded in grey, with a dotted outline). Three representative neurones are indicated by colour-coded arrows: one for each algorithm. The corresponding RFs are outlined in **(A–C)** using the corresponding colour. The STDP neurone has been chosen to illustrate a blobby RF, the ICA neurone shows a multi-lobed RF, and the SC neurone illustrates an elongated RF. Insets above and below the scatter plot show estimations of the probability density function for *n*_*x*_ and *n*_*y*_. Both axes have been cut-off at 1.5 to facilitate comparison with biological data.

As expected, units in all models converged to oriented, edge-detector like RFs. While the RFs from ICA (Figure 2B) and SC (Figure 2C) were elongated and highly directional, STDP (Figure 2A) RFs were more compact and less sharply tuned. This is closer to what is observed from simple-cell recordings in the macaque (Ringach, 2002) where RFs show high circular symmetry, and do not seem to be optimally tuned for edge-detection (see Jones and Palmer, 1987 for similar data measured in the cat). To obtain a quantitative measure of this phenomenon, we fit Gabor functions to the RFs and considered the frequency-normalised spread vectors or FSVs of the fit (Eq. 9). The first component (*n_x_*) of the FSV characterises the number of lobes in the RF, and the second component (*n_y_*) is a measure of the elongation of the RF (perpendicular to carrier propagation). A considerable number of simple-cell RFs measured in macaque tend to fall within the square bounded by *n*_*x*_ = 0.5 and *n*_*y*_ = 0.5. The FSVs of a sample of neurones (*N* = 93) measured in the macaque V1 (Ringach, 2002) indicate that 59.1% of the neurones lay within this region (Figure 2D). Since they are not elongated, and contain few lobes (typically 2–3 on/off regions), they tend to be compact – making them less effective as edge-detectors compared to more crisply tuned, elongated RFs. Amongst the three encoding schemes, while a considerable number of STDP units (82.2%) tended to fall within these realistic boundaries, ICA (10.7%) and SC (4.0%) showed a distinctive shift upwards and to the right. This trend has been observed in a number of studies using models based on ICA and SC (see, e.g., Rehn and Sommer, 2007; Puertas et al., 2010; Zylberberg et al., 2011).

The inlays in Figure 2D provide estimations of the probability densities of two FSV parameters for the macaque data and the three models. An interesting insight into these distributions is given by the KL divergence (Table 1). KL divergence (Eq. 12) is a directed measure which can be interpreted as the additional number of bits required if one of the three models were used to encode data sampled from the macaque distribution. The KL divergence for the STDP model was found to be 3.0 bits indicating that, on average, it would require three extra bits to encode data sampled from the macaque distribution. In comparison, SC and ICA were found to require 8.4 and 14.6 additional bits, respectively. An examination of the KL divergence of marginal distributions of the FSV parameters showed that STDP offers excellent encoding of both the *n_x_* (number of lobes) and the *n_y_* (compactness) parameter. ICA does not encode either of the two parameters satisfactorily, while SC performance is closer to the STDP model (especially for parameter *n_x_*).

**TABLE 1:** Kullback–Leibler (KL) divergence of the distribution of macaque frequency-normalised spread vectors (FSVs) from the models.

	**ICA**	**SC**	**STDP**
**Joint distribution**			
[*n*_*x*_ *n*_*y*_]^*T*^	14.6	8.4	3.0
**Marginal distributions**			
*n*_*x*_	7.6	1.4	1.3
*n*_*y*_	14.0	3.8	0.4

*The receptive-field (RF) shape of the neurones from the models and measurements in macaque V1 **(Ringach, 2002)** was parametrised by estimating the frequency-normalised spread vectors (FSVs). FSVs are characterised by two parameters *n*_*x*_ and *n*_*y*_: *n*_*x*_ is proportional to the number of lobes in the receptive field, and *n*_*y*_ is modulated by its elongation perpendicular to the direction of periodicity. The KL divergence reflects the number of additional bits required to encode the parameter(s) of interest from the macaque data using the distributions from one of the three models (ICA, SC, or STDP). All values are in bits.*

### Orientation Selectivity

**FIGURE 3:**
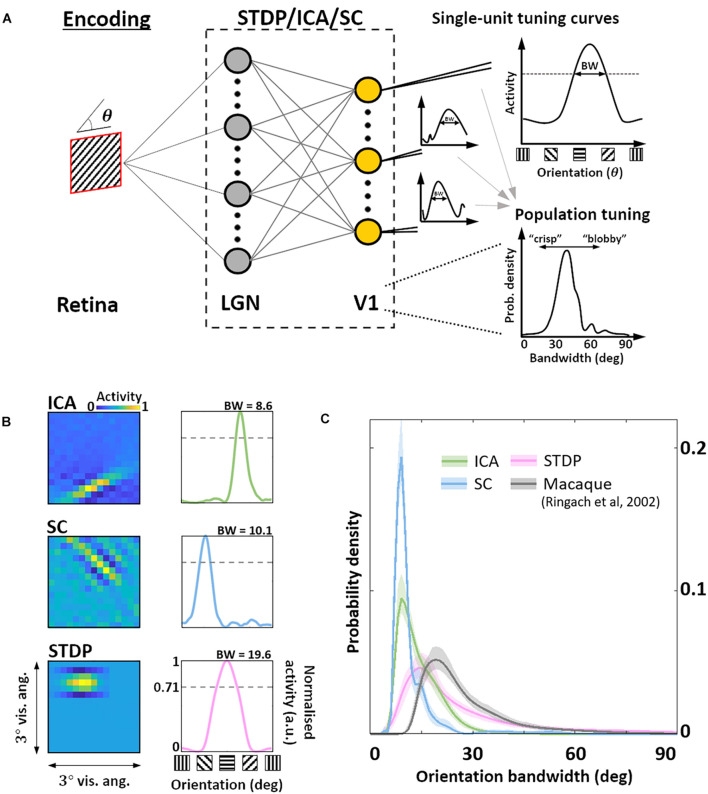
Orientation encoding. **(A)** Orientation tuning. Sine-wave gratings with additive Gaussian noise were presented to the three models to obtain single-unit orientation tuning curves (OTCs). OTC peak identifies the preferred orientation of the unit, and OTC bandwidth (half width at $1/\sqrt{2}$ peak response) is a measure of its selectivity around the peak. Low bandwidth values are indicative of sharply tuned units while high values signal broader, less specific tuning. **(B)** Single-unit tuning curves. RF (left) and the corresponding OTC (right) for representative units from ICA (top row, green), SC (second row, blue), and STDP (bottom row, pink). The bandwidth is shown above the OTC. **(C)** Population tuning. Estimated probability density of the OTC bandwidth for the three models (same colour code as panel **B**), and data measured in macaque V1 (black) **(Ringach et al., 2002)**. Envelopes around solid lines show 95% confidence intervals estimated using a bootstrap procedure. All simulations shown here were performed at an input SNR of 0 dB.

Given this sub-optimal, symmetric nature of STDP RF shapes, we next investigated how this affected the responses of these neurones to sharp edges. In particular, we were interested in how the orientation bandwidths of the units from the three models would compare to biological data. Given the RF shape, we hypothesised that orientation selectivity would be worse for STDP compared to the ICA and SC schemes. To test this hypothesis, we simulated a typical electrophysiological experiment for estimating orientation tuning (Figure 3A). To each unit, we presented noisy SWGs at its preferred spatial frequency and recorded its activity as a function of the stimulus orientation. This allowed us to plot its OTC (Figure 3B) and estimate the tuning bandwidth, which is a measure of the local selectivity of the unit around its peak – low values corresponding to sharply tuned neurones and higher values corresponding to broadly tuned, less selective neurones. For each of the three models, we estimated the pdf of the OTC bandwidth, and compared it to the distribution estimated over a large set of data (*N* = 308) measured in macaque V1 (Ringach et al., 2002) (Figure 3C). We found that ICA and SC distributions peaked at a bandwidth of about 10° (ICA: 9.1°, SC: 8.5°) while the STDP and macaque data showed much broader tunings (STDP: 15.1°, Macaque data: 19.1°). This was also reflected in the KL divergence of the macaque distribution from the three model distributions (ICA: 2.4 bits, SC: 3.5 bits, STDP: 0.29 bits). Thus, while the orientation tuning for STDP is much broader compared to ICA and SC, it is also closer to measurements in the macaque V1, indicating a better agreement with biology.

### Decoding and Information Throughput

**FIGURE 4:**
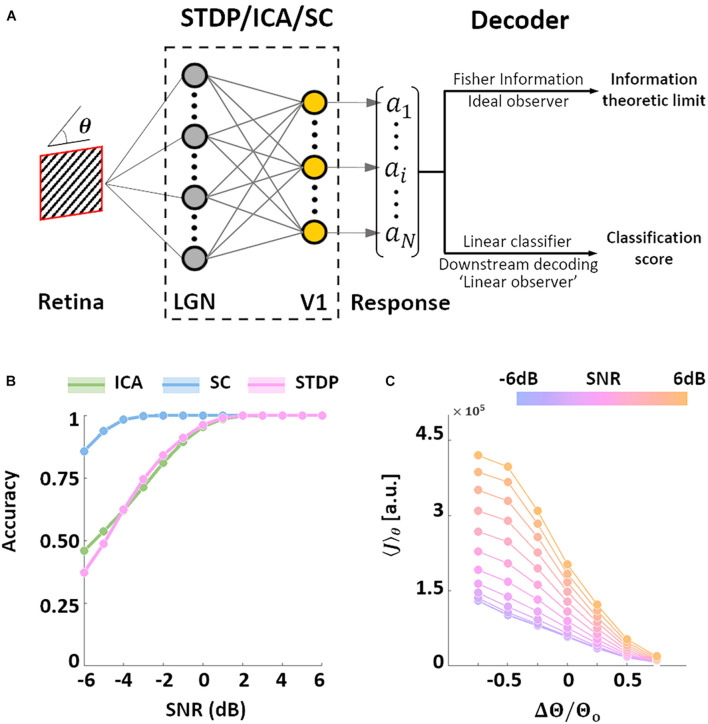
Orientation decoding. **(A)** Retrieving encoded information. Sine-wave gratings (SWGs) with varying degrees of additive Gaussian noise were presented to the three models. The following question was then posed: how much information about the stimulus (in this case, the orientation) can be decoded from the population responses? The theoretical limit of the accuracy of such a decoder can be approximated by estimating the Fisher information (FI) in the responses. In addition, a linear decoder was also used to directly decode the population responses. This could be a downstream process which is linearly driven by the population activity, or a less-than-optimal “linear observer.” **(B)** Linear decoding. The responses of each model were used to train a linear-discriminant classifier. The ordinate shows the accuracy (probability of correct classification) for each level of added noise (abscissa). Results for ICA are shown in green, SC in blue, and STDP in pink. **(C)** Post-training threshold variation in STDP. The SWG stimuli were also used to test STDP models with different values of the threshold parameter. The threshold was either increased (by 25, 50, or 75%) or decreased (by 25, 50, or 75%) with respect to the training threshold (denoted by θ_*o*_). The abscissa denotes the relative change in threshold, and the ordinate denotes the estimated FI. The colour of the lines denotes the input SNR, which ranged from −6 dB (blue) to 6 dB (orange).

After characterising the encoding capacity of the models, we next probed the possible downstream implications of such codes. The biological goal of most neural code, in the end, is the generation of behaviour that maximises evolutionary fitness. However, due to the complicated neural apparatus that separates behaviour from early sensory processing, it is not straightforward (or at times, even possible) to analyse the interaction between the two. Bearing these limitations in mind, we employed two separate metrics to investigate this relationship. In both cases, the models were presented with oriented SWGs, followed by a decoding analysis of the resulting neural population activity (Figure 4A).

We examined the performance of a decoder built on linear discriminant classifiers (these classifiers assume fixed first-order correlations in the input). Such decoders can be interpreted as linearly driven feedforward populations downstream from the thalamo-recipient layer (the “V1” populations in the three models), or a simplified, “linear” observer. Not surprisingly the accuracy of the three models increases with the SNR. We found that SC was the most accurate of the three models under all tested noise-levels, while ICA and STDP showed very similar performances (Figure 4B). SC was also more robust to Gaussian noise compared to both ICA and STDP. A major difference between the three models tested in this study is that while ICA and SC are based on linear generative units, the STDP model has an intervening thresholding nonlinearity (Eq. 6). To test the effect of this thresholding on the information throughput of the STDP model, we ran simulations where, after training on natural images, the value of the threshold parameter in the STDP model was either increased or decreased (Eq. 11). The network was presented with SWGs (same stimuli as Figure 4B), and the average FI (Eq. 10) over the orientation parameter was estimated for each simulation condition. Note that in all simulations the model was first trained (i.e., synaptic learning using natural stimuli, see Figure 1) using the same “training” threshold, and the increase/decrease of the threshold parameter was imposed post-convergence. The FI increased for thresholds lower than the training threshold – possibly driven by an increase in the overall activity of the network. On the other hand, increasing the threshold led to lower FI due to the decreased bandwidth of neural activity. Thus, it is indeed possible to manipulate the information throughput of the spiking network by regulating the overall spiking activity in the network. This trend was found to occur robustly for all tested SNR values.

## Discussion

In this study, we showed that learning in a network with an abstract, rank-based STDP rule can predict biological findings at various scales. The FSVs of the converged RFs in the model show strong similarities with single-cell data measured in the macaque primary visual cortex, while the OTCs in the model closely predict measured population tuning.

### Optimality in Biological Systems

In neuroscience, normative schemes are typically used to relate natural stimuli to an encoding hypothesis. Most normative encoding schemes optimise a generative reconstruction of the input by minimising an error metric (e.g., the L1 or L2 losses) over a given dataset. An alternative approach to studying stimulus encoding is through the use of process-based schemes which model known biophysical mechanisms at various levels of abstraction without making explicit assumptions about optimality. Traditionally, process-based or mechanistic schemes do not employ error metrics, and have been used to study fine-grained neuronal dynamics (Kang and Sompolinsky, 2001; Moreno-Bote et al., 2014; Harnack et al., 2015). On the other hand, normative schemes are employed to describe population-level characteristics (Olshausen and Field, 1997; van Hateren and van der Schaaf, 1998; Lee and Seung, 1999; Hoyer and Hyvärinen, 2000). In this study, we show that RFs predicted by a non-generative rank-based STDP rule are closer to electrophysiological measurements in the macaque V1 when compared to generatively optimal schemes such as ICA and SC. While this study only employs the classical variations of ICA and SC, subsequent work has demonstrated that similar suboptimalities in RF shape can also be obtained by generative models when biologically plausible nonlinearities such as thresholding operations (Rehn and Sommer, 2007; Rozell et al., 2008), or pointwise maxima operations (Puertas et al., 2010) are introduced. However, the abstract rank-based STDP model used here is free from generative optimisation and offers a much more biologically plausible, normative description of “learning” through experience in the early visual system, where there is no sensory “ground truth” to generate errors from.

Note that while process-based models can predict suboptimalities observed in biological data, they cannot account for the theoretical insights offered by generative normative schemes. Local synaptic processes such as STDP can, in fact, be viewed as neural substrates for the overall synaptic optimisation employed by these schemes. The critique that gradient descent is inherently biologically implausible is being challenged by recent studies which frame error propagation and stochastic descent in terms of local, biologically plausible rules (see, e.g., Lillicrap et al., 2016; Melchior and Wiskott, 2019; Li, 2020). It has been demonstrated that local plasticity rules can, in fact, be adapted to describe various normative hypotheses about stimulus encoding (Savin et al., 2010; Brito and Gerstner, 2016). A growing number of insightful studies now employ hybrid encoding schemes which address multiple optimisation criteria (Perrinet and Bednar, 2015; Martinez-Garcia et al., 2017; Beyeler et al., 2019), often through local biologically realistic computation (Rozell et al., 2008; Savin et al., 2010; Zylberberg et al., 2011; Isomura and Toyoizumi, 2018).

### Sparsity

**FIGURE 5:**
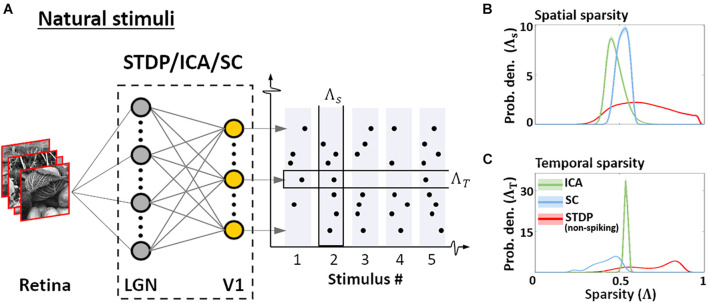
Sparsity. **(A)** Sparsity indices. To estimate the sparsity of the non-spiking responses to natural stimuli, 10^4^ patches (3° × 3° visual angle) randomly sampled from natural scenes were presented to the three models. Two measures of sparsity were defined: Spatial sparsity Index (Λ_*S*_) was defined as the average sparsity of the activity of the entire neuronal ensemble, while Temporal sparsity Index (Λ_*T*_) was defined as the average sparsity of the activity of single neurones to the entire input sequence. **(B)** Spatial sparsity. Estimated probability density of Λ_*S*_ for ICA (green), Sparse Coding (blue), and STDP (red). Λ_*S*_ varied between 0 (all units activate with equal intensity) and 1 (only 1 u/U activates) by definition. **(C)** Temporal sparsity. Estimated probability density of Λ_*T*_, shown in a manner analogous to Λ_*S*_ (panel **B**). Λ_*T*_ also varied between 0 (homogeneous activity for the entire input sequence) and 1 (activity only for few inputs in the sequence).

Normative descriptions of the early visual system are grounded in the idea of efficiency – in terms of information transfer, and in terms of resource consumption. These assumptions, in turn, determine the behaviour of population responses to natural images. We quantified this behaviour by presenting the converged models with patches randomly sampled from the training dataset of natural images, and estimating the sparsity of the resulting activations using the Gini coefficient (Eq. 13; Hurley and Rickard, 2009). The sparsity was examined in two contexts (Barth and Poulet, 2012) as shown in Figure 5A. First, sparsity of the entire ensemble was estimated for each presented stimulus – this is a measure of how many neurones, on average, are employed by the ensemble to encode a given stimulus (Eq. 14). Second, the sparsity of individual neurones over the entire sequence of stimuli was estimated, allowing us to infer how frequently the features selected/encoded by the converged models occur in the sequence (Eq. 15). We denote the former as spatial sparsity (Λ_*s*_), and the latter as temporal sparsity (Λ_*t*_). For STDP, the indices were calculated for the membrane potential to facilitate comparison with ICA and SC activations. STDP membrane potential (red, Figure 5B) shows high variability in Λ_*s*_, whereas ICA (green) and SC (blue) show much lower variance in comparison. This suggests that ICA and SC converge to features such that each image activates approximately equal number of units. On the other hand, the sparsity of the STDP neurones is more variable and stimulus-dependent, and likely driven by the relative probability of occurrence of specific features in the dataset – thus reflecting the Hebbian principal. ICA also exhibits a similar, small range for temporal sparsity Λ_*t*_ (Figure 5C) – suggesting that ICA encoding has uniform activation probability across its units. SC and STDP, however, show a much broader range of temporal sparsity across their units, with some units activating more frequently as compared to others.

Taken together, this suggests that the ICA encoding scheme converges to features such that the activation is distributed uniformly across the units, both for a given stimulus, and across multiple stimuli. This is likely to be driven by the objective of minimising reconstruction loss while maintaining minimal mutual information across the population. SC, on the other hand, equalises the probability of firing over the population for any given stimulus, but individual units may converge to features which occur more or less frequently. Once again, this behaviour is a consequence of the loss function which ensures that the network activity is sparse for each stimulus, but does not impose explicit constraints between the activity profile of individual units. As the STDP model is unsupervised and does not explicitly impose any generative loss function, we find high variability in both the spatial and temporal sparsity of STDP units. As shown in Figure 4C, this variability ensures that the information throughput of the network can be modulated by regulation of parameters such as the spiking threshold, even after the initial training.

### Emerging Technologies and Process-Based Modelling in Neuroscience

Traditionally, detailed process-based models have suffered from constraints imposed by computational complexity, prohibitively long execution times which do not scale well for large networks, and hardware that is geared toward synchronous processing. On the other hand, most normative models can leverage faster computational libraries and architectures which have been developed over several decades, thereby leading to more efficient and scalable computation. However, with the growing availability of faster and more adaptable computing solutions such as neuromorphic hardware (event-based cameras, spike-based chips), and event-driven computational frameworks (e.g., Nengo: Bekolay et al., 2014; or Brian 2: Stimberg et al., 2019), implementations of such models are becoming increasingly accessible and scientifically tractable. These frameworks can be used not only to investigate detailed biophysical models or create biologically relevant machine and reinforcement learning pipelines, but to also investigate normative neuroscientific hypotheses which require unsupervised learning. In the future, we hope process-based modelling will be adopted more widely by cognitive and computational neuroscientists alike.

## Data Availability Statement

Publicly available datasets were analysed in this study. This data can be found here: http://ringachlab.net/.

## Author Contributions

TC, BC, and TM conceptualised the study and interpreted the results. TC designed, programmed, and ran the simulations and wrote the original draft. All authors reviewed and revised the manuscript.

## Conflict of Interest

The authors declare that the research was conducted in the absence of any commercial or financial relationships that could be construed as a potential conflict of interest.

## Publisher’s Note

All claims expressed in this article are solely those of the authors and do not necessarily represent those of their affiliated organizations, or those of the publisher, the editors and the reviewers. Any product that may be evaluated in this article, or claim that may be made by its manufacturer, is not guaranteed or endorsed by the publisher.

## References

[B1] Anderson C. Van Essen D. Olshausen B. (2005). “CHAPTER 3 - Directed visual attention and the dynamic control of information flow,” in *Neurobiology of Attention*, eds Itti L. Rees G. Tsotsos J. K. (Burlington, VT: Academic Press), 11–17. 10.1016/B978-012375731-9/50007-0

[B2] Barth A. L. Poulet J. F. A. (2012). Experimental evidence for sparse firing in the neocortex. *Trends Neurosci.* 35 345–355. 10.1016/j.tins.2012.03.008 22579264

[B3] Bekolay T. Bergstra J. Hunsberger E. DeWolf T. Stewart T. Rasmussen D. (2014). Nengo: a Python tool for building large-scale functional brain models. *Front. Neuroinformatics* 7:48. 10.3389/fninf.2013.00048 24431999 PMC3880998

[B4] Bell A. Sejnowski T. (1997). The “independent components” of natural scenes are edge filters. *Vision Res.* 37 3327–3338. 10.1016/S0042-6989(97)00121-1 9425547 PMC2882863

[B5] Beyeler M. Rounds E. Carlson K. Dutt N. Krichmar J. (2019). Neural correlates of sparse coding and dimensionality reduction. *PLoS Comput. Biol.* 15:e1006908. 10.1371/journal.pcbi.1006908 31246948 PMC6597036

[B6] Brito C. S. N. Gerstner W. (2016). Nonlinear Hebbian learning as a unifying principle in receptive field formation. *PLoS Comput. Biol.* 12:e1005070. 10.1371/journal.pcbi.1005070 27690349 PMC5045191

[B7] Bruce N. Rahman S. Carrier D. (2016). Sparse coding in early visual representation: from specific properties to general principles. *Neurocomputing* 171 1085–1098. 10.1016/j.neucom.2015.07.070

[B8] Caporale N. Dan Y. (2008). Spike timing–dependent plasticity: a Hebbian learning rule. *Annu. Rev. Neurosci.* 31 25–46. 10.1146/annurev.neuro.31.060407.125639 18275283

[B9] Chauhan T. Masquelier T. Montlibert A. Cottereau B. (2018). Emergence of binocular disparity selectivity through Hebbian learning. *J. Neurosci.* 38 9563–9578. 10.1523/JNEUROSCI.1259-18.2018 30242050 PMC6705998

[B10] Delorme A. Perrinet L. Thorpe S. J. (2001). Networks of integrate-and-fire neurons using rank order coding B: spike timing dependent plasticity and emergence of orientation selectivity. *Neurocomputing* 38–40 539–545. 10.1016/S0925-2312(01)00403-9

[B11] Ecke G. A. Papp H. M. Mallot H. A. (2021). Exploitation of image statistics with sparse coding in the case of stereo vision. *Neural Netw.* 135 158–176. 10.1016/j.neunet.2020.12.016 33388507

[B12] Geisler W. (2008). Visual perception and the statistical properties of natural scenes. *Annu. Rev. Psychol.* 59 167–192. 10.1146/annurev.psych.58.110405.085632 17705683

[B13] Gütig R. Aharonov R. Rotter S. Sompolinsky H. (2003). Learning input correlations through nonlinear temporally asymmetric Hebbian plasticity. *J. Neurosci.* 23 3697–3714. 12736341 10.1523/JNEUROSCI.23-09-03697.2003 PMC6742165

[B14] Harnack D. Pelko M. Chaillet A. Chitour Y. van Rossum M. C. W. (2015). Stability of neuronal networks with homeostatic regulation. *PLoS Comput. Biol.* 11:e1004357. 10.1371/journal.pcbi.1004357 26154297 PMC4495932

[B15] Hoyer P. Hyvärinen A. (2000). Independent component analysis applied to feature extraction from colour and stereo images. *Netw. Comput. Neural Syst.* 11 191–210. 10.1088/0954-898X_11_3_302 11014668

[B16] Hübener M. Bonhoeffer T. (2014). Neuronal plasticity: beyond the critical period. *Cell* 159 727–737. 10.1016/j.cell.2014.10.035 25417151

[B17] Hunter D. Hibbard P. (2015). Distribution of independent components of binocular natural images. *J. Vis.* 15:6. 10.1167/15.13.6 26381837

[B18] Hurley N. Rickard S. (2009). Comparing measures of sparsity. *IEEE Trans. Inf. Theory* 55 4723–4741. 10.1109/TIT.2009.2027527

[B19] Hyvärinen A. Oja E. (2000). Independent component analysis: algorithms and applications. *Neural Netw.* 13 411–430. 10.1016/S0893-6080(00)00026-5 10946390

[B20] Isomura T. Toyoizumi T. (2018). Error-gated Hebbian rule: a local learning rule for principal and independent component analysis. *Sci. Rep.* 8:1835. 10.1038/s41598-018-20082-0 29382868 PMC5789861

[B21] Jones J. Palmer L. (1987). An evaluation of the two-dimensional Gabor filter model of simple receptive fields in cat striate cortex. *J. Neurophysiol.* 58 1233–1258. 3437332 10.1152/jn.1987.58.6.1233

[B22] Kang K. Sompolinsky H. (2001). Mutual Information of population codes and distance measures in probability space. *Phys. Rev. Lett.* 86 4958–4961. 10.1103/PhysRevLett.86.4958 11384391

[B23] Larsen R. S. Rao D. Manis P. B. Philpot B. D. (2010). STDP in the developing sensory neocortex. *Front. Synaptic Neurosci.* 2:9. 10.3389/fnsyn.2010.00009 21423495 PMC3059680

[B24] Lee D. Seung H. (1999). Learning the parts of objects by non-negative matrix factorization. *Nature* 401 788–791. 10.1038/44565 10548103

[B25] Li H. L. (2020). Faster biological gradient descent learning. *ArXiv* [Preprint] ArXiv 200912745 Cs,

[B26] Lillicrap T. P. Cownden D. Tweed D. B. Akerman C. J. (2016). Random synaptic feedback weights support error backpropagation for deep learning. *Nat. Commun.* 7:13276. 10.1038/ncomms13276 27824044 PMC5105169

[B27] Markram H. Lübke J. Frotscher M. Sakmann B. Hebb D. O. Bliss T. V. P. (1997). Regulation of synaptic efficacy by coincidence of postsynaptic APs and EPSPs. *Science* 275 213–215. 10.1126/science.275.5297.213 8985014

[B28] Martinez-Garcia M. Martinez L. M. Malo J. (2017). Topographic Independent Component Analysis reveals random scrambling of orientation in visual space. *PLoS One* 12:e0178345. 10.1371/journal.pone.0178345 28640816 PMC5480835

[B29] Masquelier T. (2012). Relative spike time coding and STDP-based orientation selectivity in the early visual system in natural continuous and saccadic vision: a computational model. *J. Comput. Neurosci.* 32 425–441. 10.1007/s10827-011-0361-9 21938439

[B30] Masquelier T. Thorpe S. J. (2007). Unsupervised learning of visual features through spike timing dependent plasticity. *PLoS Comput. Biol.* 3:e31. 10.1371/journal.pcbi.0030031 17305422 PMC1797822

[B31] Melchior J. Wiskott L. (2019). Hebbian-Descent. *ArXiv* [Preprint] ArXiv190510585 Cs Stat. Available online at: http://arxiv.org/abs/1905.10585 [Accessed August 20, 2021],

[B32] Moreno-Bote R. Beck J. Kanitscheider I. Pitkow X. Latham P. Pouget A. (2014). Information-limiting correlations. *Nat. Neurosci.* 17 1410–1417. 10.1038/nn.3807 25195105 PMC4486057

[B33] Olshausen B. Field D. (1996). Emergence of simple-cell receptive field properties by learning a sparse code for natural images. *Nature* 381 607–609. 10.1038/381607a0 8637596

[B34] Olshausen B. Field D. (1997). Sparse coding with an overcomplete basis set: a strategy employed by V1? *Vision Res.* 37 3311–3325. 10.1016/S0042-6989(97)00169-7 9425546

[B35] Perrinet L. U. Bednar J. A. (2015). Edge co-occurrences can account for rapid categorization of natural versus animal images. *Sci. Rep.* 5:11400. 10.1038/srep11400 26096913 PMC4476147

[B36] Puertas J. Bornschein J. Lücke J. (2010). “The maximal causes of natural scenes are edge filters,” in *Advances in Neural Information Processing Systems*, eds Lafferty J. D. Williams C. K. I. Shawe-Taylor J. Zemel R. S. Culotta A. (Red Hook, NY: Curran Associates, Inc), 1939–1947.

[B37] Raichle M. (2010). Two views of brain function. *Trends Cogn. Sci.* 14 180–190. 10.1016/j.tics.2010.01.008 20206576

[B38] Rehn M. Sommer F. (2007). A network that uses few active neurones to code visual input predicts the diverse shapes of cortical receptive fields. *J. Comput. Neurosci.* 22 135–146. 10.1007/s10827-006-0003-9 17053994

[B39] Ringach D. (2002). Spatial structure and symmetry of simple-cell receptive fields in Macaque primary visual cortex. *J. Neurophysiol.* 88 455–463. 12091567 10.1152/jn.2002.88.1.455

[B40] Ringach D. Shapley R. (2004). Reverse correlation in neurophysiology. *Cogn. Sci.* 28 147–166. 10.1207/s15516709cog2802_2

[B41] Ringach D. Shapley R. Hawken M. (2002). Orientation selectivity in Macaque V1: diversity and laminar dependence. *J. Neurosci.* 22 5639–5651. 10.1523/JNEUROSCI.22-13-05639.2002 12097515 PMC6758222

[B42] Rozell C. Johnson D. Baraniuk R. Olshausen B. (2008). Sparse coding via thresholding and local competition in neural circuits. *Neural Comput.* 20 2526–2563. 10.1162/neco.2008.03-07-486 18439138

[B43] Savin C. Joshi P. Triesch J. (2010). Independent component analysis in spiking neurons. *PLoS Comput. Biol.* 6:e1000757. 10.1371/journal.pcbi.1000757 20421937 PMC2858697

[B44] Stimberg M. Brette R. Goodman D. F. (2019). Brian 2, an intuitive and efficient neural simulator. *eLife* 8:e47314. 10.7554/eLife.47314 31429824 PMC6786860

[B45] van Hateren J. van der Schaaf A. (1998). Independent component filters of natural images compared with simple cells in primary visual cortex. *Proc. Biol. Sci.* 265 359–366. 10.1098/rspb.1998.0303 9523437 PMC1688904

[B46] Wandell B. Smirnakis S. (2010). Plasticity and stability of visual field maps in adult primary visual cortex. *Nat. Rev. Neurosci.* 10:873. 10.1038/nrn2741 19904279 PMC2895763

[B47] Zhaoping L. (2006). Theoretical understanding of the early visual processes by data compression and data selection. *Netw. Comput. Neural Syst.* 17 301–334. 10.1080/09548980600931995 17283516

[B48] Zylberberg J. Murphy J. T. DeWeese M. R. (2011). A Sparse coding model with synaptically local plasticity and spiking neurons can account for the diverse shapes of V1 simple cell receptive fields. *PLoS Comput. Biol.* 7:e1002250. 10.1371/journal.pcbi.1002250 22046123 PMC3203062

